# Isolation and Functional Analysis of *EPHEMERAL1-LIKE* (*EPH1L*) Genes Involved in Flower Senescence in Cultivated Japanese Gentians

**DOI:** 10.3390/ijms23105608

**Published:** 2022-05-17

**Authors:** Shigekazu Takahashi, Chiharu Yoshida, Hideyuki Takahashi, Masahiro Nishihara

**Affiliations:** 1 Iwate Biotechnology Research Center, Kitakami 024-0003, Japan; s-takahashi@ibrc.or.jp (S.T.); chiharu-y@ibrc.or.jp (C.Y.); 2 Department of Agriculture, School of Agriculture, Tokai University, Kumamoto 862-8652, Japan; takahashi.hideyuki.m@tokai.ac.jp

**Keywords:** corolla, CRISPR/Cas9, *EPHEMERAL1*, flower longevity, genome editing, Japanese gentian, NAC transcription factor, senescence

## Abstract

The elongation of flower longevity increases the commercial value of ornamental plants, and various genes have been identified as influencing flower senescence. Recently, *EPHEMERAL1* (*EPH1*), encoding a NAC-type transcription factor, was identified in Japanese morning glory as a gene that promotes flower senescence. Here we attempted to identify an *EPH1* homolog gene from cultivated Japanese gentians and characterized the same with regard to its flower senescence. Two *EPH1*-*LIKE* genes (*EPH1La* and *EPH1Lb*), considered as alleles, were isolated from a gentian cultivar (*Gentiana scabra* × *G. triflora*). Phylogenetic analyses revealed that EPH1L belongs to the NAM subfamily. The transcript levels of *EPH1L* increased along with its senescence in the field-grown flowers. Under dark-induced senescence conditions, the gentian-detached flowers showed the peak transcription level of *EPH1L* earlier than that of *SAG12*, a senescence marker gene, suggesting the involvement of *EPH1L* in flower senescence. To reveal the *EPH1L* function, we produced *eph1l*-knockout mutant lines using the CRISPR/Cas9 system. When the flower longevity was evaluated using the detached flowers as described above, improved longevity was recorded in all genome-edited lines, with delayed induction of *SAG12* transcription. The degradation analysis of genomic DNA matched the elongation of flower longevity, cumulatively indicating the involvement of *EPH1L* in the regulation of flower senescence in gentians.

## 1. Introduction

Ornamental flowers contribute to the quality of human life in numerous situations; they are sometimes used as display items, presents, or decorations for streets or homes. As a special case, a specific floral arrangement has been used for improving the visuospatial working memory of schizophrenic patients, which makes ornamental flowers important in the medical field as well [1]. Nowadays, there are innumerable types of flowers available that are used for ornamental purposes, and novel varieties with different traits are being continuously developed and placed on the market. The value of ornamental flowers in the market is determined by various traits, such as their color, shape, and scent, based on the consumers’ requirements. However, these traits are diverse and applicable depending on the usage and personal preference, as there is no common requirement for such flowers. On the other hand, flower longevity is an important trait commonly applicable to all ornamental plants. The longer the flower longevity can be extended for the viewing period, the more pleasing it is to the consumers. In addition, it is noteworthy that prolonging a flower’s life offers great advantage in terms of long-term storage, enabling low-loss transportation through sales channels from producers to retailers. There exist various definitions of flower senescence, including wilting, abscission, color fading by the effects of aging, abiotic and biotic stresses, and pollination, but here we are referring to petal (including corolla) senescence caused by aging. Against this background, numerous studies on flower senescence have been conducted so far [2].

Flower senescence is linked to a typical programmed cell death (PCD), which is highly regulated according to the flower’s developmental stage [3,4,5]. Furthermore, flower senescence is mainly divided into two types: ethylene-dependent and ethylene-independent. In the former type, the increase in endogenous ethylene production triggers petal senescence [6,7]. Treatment with silver thiosulfate (STS), which inhibits ethylene signaling, can extend a flower’s life and become the most-used method for ethylene-sensitive flowers. On the other hand, in the latter type, the amount of endogenous ethylene is small, and the senescence of flowers is rarely promoted by treatment with exogenous ethylene [2]. Ethylene-mediated flower senescence has been very well studied, and the biosynthetic genes and signaling pathways involved in this event are also well understood [2]. On the other hand, the molecular mechanism that regulates flower senescence (i.e., petal PCD) by an ethylene-independent pathway remains unknown. Recently, a novel gene named *EPHEMRAL1* (*EPH1*) that encodes a NAC (NAM/ATAF1, 2/CUC2)-type transcription factor was identified in Japanese morning glory as the key regulator of PCD in petal senescence [8,9]. *EPH1* is also involved in the regulation of ethylene-accelerated petal senescence. In fact, *EPH1* is undoubtedly considered to be the key gene that accelerates flower senescence, because flower longevity can be prolonged by suppressing or disrupting *EPH1* by using RNAi and genome-editing technology [8,9]. However, the role of *EPH1* in other plant species remains unclear.

Japanese cultivated gentian is one of the major ornamental plants in Japan, used as cut flowers and potted flowers. To date, >300 gentian cultivars have been bred and used in the market [10]. These gentian cultivars are derived from *Gentiana scabra*, *G. triflora*, and their hybrids. Studies on various traits of Japanese gentian flowers (e.g., color [11,12], flower shape [13,14], flowering time [15], and floral odor [16]) have been performed so far. Furthermore, in recent years, knowledge about the metabolic pathway of flower colors in Japanese gentian has accumulated through molecular biology techniques [17,18,19,20,21,22]. In addition, the development of DNA markers to distinguish the flower color and shape is progressing based on these findings [23,24,25]. Recently, it was revealed that Japanese gentian performs photosynthesis at the green spots on the corolla lobe [26]. Aquaporins involved in gentian flower opening have been also revealed [27]. Photosynthesis occurring in the corolla and the movement of the flower while opening and closing may affect flower longevity; however, detailed analyses regarding flower senescence have not been performed. In these experiments, biological tools, such as virus-induced gene silencing and genome editing, have been developed and found helpful in characterizing the gene functions in gentians [13,21,22,27]. Among studies conducted on the effect of ethylene treatment against senescence, it has been reported that *G. scabra* is highly sensitive [28], while other gentians (*G. dahurica*, *G. kochiana*, *G. sino-ornata* [29], and *G. triflora* [30]) are less sensitive. In addition, Zhang and Leung (2001) demonstrated that pulsed treatment with STS could prolong the longevity of cut flowers of *G. triflora* [31]. Although the effects of ethylene treatment on gentian flowers have been reported, no genes responsible for flower senescence, including *EPH1*, have yet been identified in gentians; therefore, the regulation of flower senescence remains unknown at the molecular level.

Here, we first isolated *EPEMERAL1-LIKE* (*EPH1L*), which shares homology to morning glory *EPH1*, from a Japanese gentian and analyzed its involvement in flower senescence by using the CRISPR/Cas9 genome-editing technology. For this purpose, we developed a highly reproducible evaluation system for flower senescence using detached flowers under dark-induced senescence conditions and analyzed the delayed flower senescence, including genomic DNA degradation and the expression levels of senescence-associated genes. As a result, improved flower longevity was confirmed in three independent *eph1l*-knockout mutant lines, suggesting that *EPH1L* is undoubtedly involved in flower senescence in gentians. Our study also demonstrates that *eph1l*-edited mutant lines are useful as breeding materials in the gentian-breeding program, and that this strategy can be applied to other ornamental flowers.

## 2. Results and Discussion

### 2.1. Identification of a Homologous Gene of Morning Glory EPHEMERAL1 (EPH1) in Gentians

**Figure 1:**
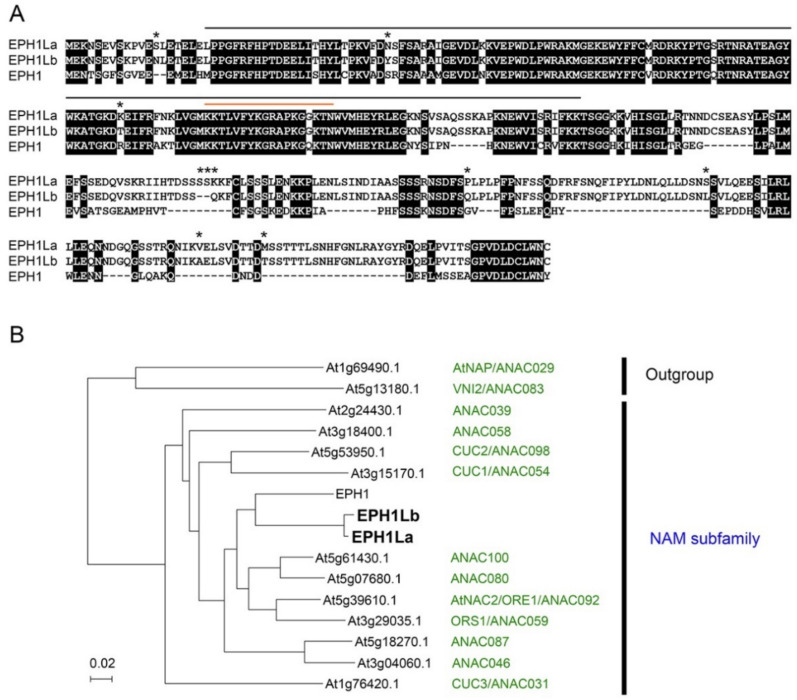
(**A**) Alignment of the deduced amino acid sequences of EPH1 and EPH1L proteins. The amino acid sequences of EPH1 from the Japanese morning glory (*Ipomoea nil*) and EPH1L (EPH1La and EPH1Lb) from the Japanese gentian (“Albireo”) were aligned by using the Clustal X program [32]. The amino acid residues conserved among the proteins are highlighted in black. The amino acid residues that are different between EPH1La and EPH1Lb are indicated with an asterisk *. The black and orange overlines indicate the conserved NAC domain and the putative nuclear localization signal, respectively. Asterisks *** indicate different amino acid residues in EPH1La and EPH1Lb. (**B**) The molecular phylogenic tree of EPH1, EPH1La, EPH1Lb, and *Arabidopsis* NAC proteins belonging to the NAM subfamily. The branch lengths were proportional to the genetic distances as calculated by the neighbor-joining method [33]. Amino acid sequences for all NAM subfamilies and outgroups in *A. thaliana* were obtained via TAIR (http://www.arabidopsis.org/, accessed on 1 April 2022).

First, we searched for a gene homologous to *EPH1* using the RNA-seq database of the “Hakuju” (accession no. DRA012949), which is one of the Japanese gentian cultivars, and found a candidate sequence (TRINITY_DN36649_c0_g1_i1) that shared a high homology with *EPH1* (Figure S1). Based on this sequence, we attempted to clone a gene from “Albireo,” which is another gentian cultivar that is easy to transform to produce the knockout mutant lines by CRISPR/Cas9-mediated genome editing. When we applied the “Albireo” cDNA as a template, the coding region sequences of different sizes of 1104 bp and 1095 bp were obtained, which were named as the sequences *EPHEMERAL1-LIKEa* (*EPH1La*) and *EPHEMERAL1-LIKEb* (*EPHL1b*), respectively. Subsequently, we cloned the genomic sequences of *EPH1La* and *EPH1Lb* and found that both the genes were comprised of three exons and two introns (Figure S2). EPH1La and EPH1Lb showed 97.8% amino acid identity (Figure 1A). These sequences are available in the public database under accession nos. LC703152 and LC703153. Because “Albireo” is a hybrid of *G. triflora* and *G. scabra*, *EPH1La* and *EPH1Lb* are considered allelic to each other despite their different sequence sizes. The cDNA of *EPH1Lb* is the same as TRINITY_DN36649_c0_g1_i1 derived from “Hakuju”, which is *G. scabra*, and *EPH1La* is likely derived from *G. triflora*. Hereafter, we have described them together as *EPH1L*. The deduced amino acid sequences of the gentian EPH1L and the morning glory EPH1 are aligned (Figure 1A). EPH1L and EPH1 are highly conserved at the N-terminal side, including the NAC domain and the putative nuclear localization signal, albeit the structure is different toward the C-terminal side, with EPH1L being larger (Figure 1A). EPH1 belongs to the NAM subfamily of the NAC family [8]. Figure 1B demonstrates the molecular phylogenetic tree of the 11 genes belonging to the NAM subfamily of *Arabidopsis thaliana*, EPH1, and EPH1L. EPH1L also belongs to the NAM subfamily and is deemed closer to EPH1 than to the *Arabidopsis* NAM subfamily.

### 2.2. Transcription of EPH1L Is Induced with Senescence in Field-Grown Gentians

**Figure 2:**
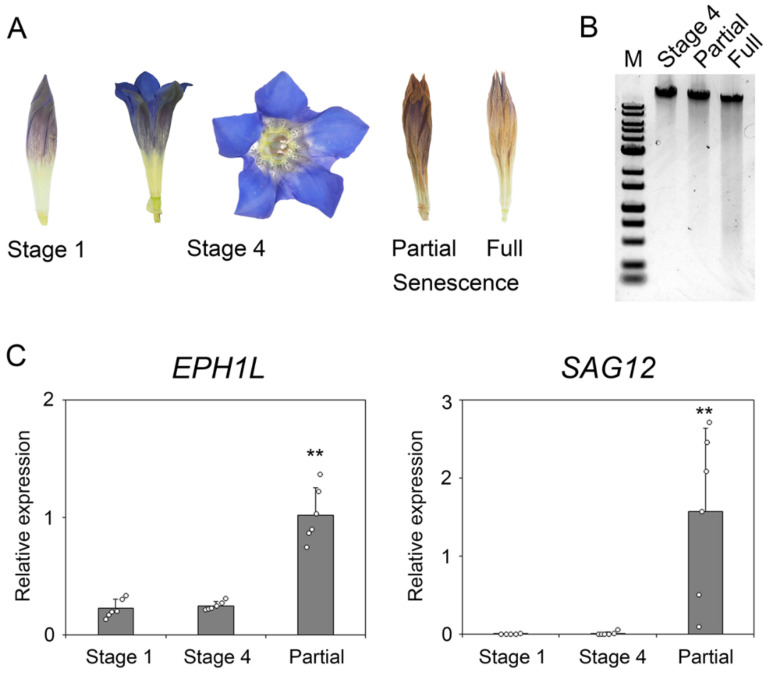
Changes in the corolla appearance, genome structure, and gene expression with aging of field-grown Japanese gentians. (**A**) The appearance of field-grown Japanese gentians (“Albireo”). (**B**). Image of the agarose gel electrophoresis for the “Albireo” corolla genome at stage 4 and senescence corolla (partially wilted and fully wilted). (**C**) The relative expression level of *EPH1L* and *SAG12* determined by a quantitative reverse transcription polymerase chain reaction (*n* = 6). Asterisks indicate statistically significant differences compared with stage 1 as demonstrated by Student’s *t*-test. (** *p* < 0.01).

Next, we investigated the alterations in the flower’s appearance and damage (such as the degradation of genomic DNA) during flower senescence using flower samples grown under a natural environment (Figure 2A,B). Accordingly, we prepared two types of aging samples, one with the top of the corolla browned and the other with the entire corolla browned. The corolla developmental stages (S1–S4) were designated as described by Nakatsuka et al. [17]. When the genomic DNAs extracted from stage 4 (fully opened flowers) and two types of aged flowers were subjected to agarose gel electrophoresis, degradation was noted depending on the degree of senescence (Figure 2B). This phenomenon was also reported in a previous study on Japanese morning glory [8]; therefore, it is believed that the mechanism of genomic DNA degradation by PCD associated with aging also exists in gentian flowers and is considered as an indicator of flower senescence.

In order to clarify whether the *EPH1L* expression is induced during senescence in gentian corolla under natural environmental conditions, the transcription levels of *EPH1L* in the corolla of different stages of field-grown gentian plants were determined by quantitative reverse transcription polymerase chain reaction (qRT-PCR) (Figure 2C). The transcriptional levels of *EPH1L* and a senescence marker gene, a gentian homolog of a senescence-associated gene (*SAG12*, accession no. LC707747), which encodes a cysteine protease, were found to be higher in the corolla with a brown apex when compared to the corolla of stages 1 and 4 that were not aging. As there was no significant difference in the *EPH1L* transcriptional level between stage 1 and stage 4, we considered that the induction of *EPH1L* expression in the field occurred after developmental stage 4.

### 2.3. Establishment of a Reliable Evaluation System for Flower Longevity of Gentians Using Dark-Induced Senescence

**Figure 3:**
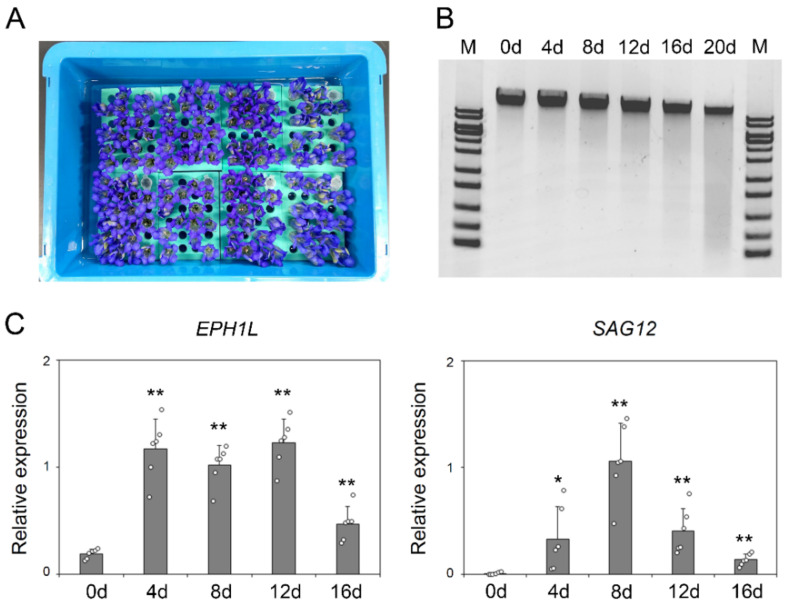
Dark-induced senescence of “Albireo” corolla at stage 4. (**A**) The state of “Albireo” corolla before the start of dark-induced senescence treatment. The cover is opened for the photograph. (**B**) Image of the agarose gel electrophoresis of the genomic DNAs of “Albireo” corolla from before the start of the dark-induced senescence treatment until the 20th day after the start of the treatment (every 4 days). (**C**) The relative expression levels of *EPH1L* and *SAG12* determined by quantitative reverse transcription polymerase chain reaction (*n* = 6). Asterisks indicate statistically significant differences between 0 d and 4–16 d as demonstrated by Student’s *t*-test (* *p* < 0.05, ** *p* < 0.01).

The flower longevity of gentians is usually more than 1 week, which is overwhelmingly longer than that of Japanese morning glory, which have 1-day of lifespan, and it is quite difficult to evaluate the senescence quantitatively. Namely, the environment conditions, such as temperature and light, drastically change in the field, and flower longevity varies according to the weather. Thus, the examination of the exact expression fluctuation of *EPH1L* with senescence is difficult in the actual field. Instead, a reproducible experimental system for flower senescence is warranted to investigate the details of the transcriptional level of *EPH1L* in association with senescence. For evaluating leaf senescence, a dark-induced senescence treatment has been widely used [34]. In this study, we examined whether this method was applicable to evaluate flower longevity in gentians. From the above-mentioned results, as the expression of *EPH1L* in field-grown corolla occurs after stage 4, the corollas of stage 4 were collected and subjected to dark-induced senescence as shown in Figure 3A. Sampling was performed every 4 days, with the degradation levels of the corolla genomic DNAs being analyzed by agarose gel electrophoresis. As a result, the genome degradation levels of fully wilted corolla could be mimicked by dark treatment for 20 days (Figure 3B). Subsequently, the expression fluctuations of *EPH1L* and *SAG12* were analyzed by qRT-PCR (Figure 3C). The transcription level of *EPH1L* peaked on the 4th day from the start of the dark treatment and was maintained at the same level until the 12th day. On the other hand, the peak of *SAG12* was detected on the 8th day from the start of the dark treatment. The induction of *EPH1L* expression prior to the *SAG12* expression was found to be similar to the expression profile of *EPH1* and *SAG12* in Japanese morning glory [8], and it was considered that gentian *EPH1L* was involved in flower longevity as well as *EPH1*.

### 2.4. Production and Sequence Analyses of eph1l Genome-Edited Gentian Lines

**Figure 4:**
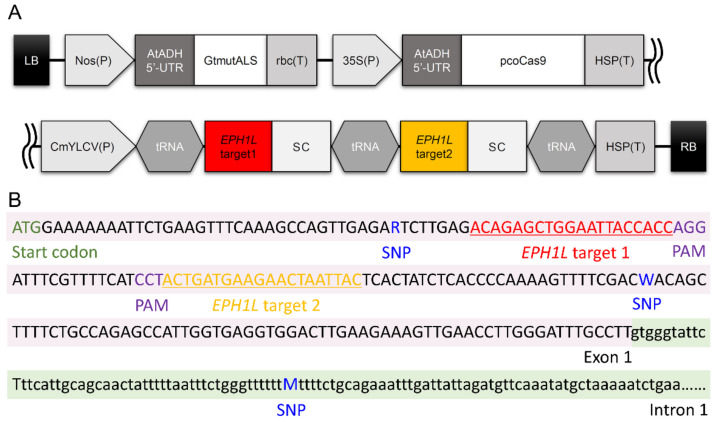
Schematic diagram of the binary vector and the target sequences in the *EPHEMERAL1-LIKE* in Japanese gentians. (**A**) The diagram of the pSALS-35SpCas9-CmYLCVpEPH1Lt1t2. LB, left border of T-DNA; Nos(P), promoter of the nopaline synthase gene of *Agrobacterium tumefaciens*; AtADH 5′-UTR, 5′—untranslated region of the alcohol dehydrogenase gene of *Arabidopsis thaliana*; GtmutALS, mutant acetolactate synthase gene of *Gentiana triflora*; rbc(T), terminator of small subunit 2B of ribulose 1, 5-bisphosphate carboxylase/oxygenase of *A. thaliana*; 35SP(P), Cauliflower mosaic virus 35S promoter; pcoCas9, plant codon-optimized Cas9 of *Streptococcus pyogenes*; HSP(T), terminator of heat shock protein 18.2 of *A. thaliana*; CmYLCV(P), Cestrum yellow leaf curling virus promoter; SC, single-guide RNA scaffold; RB, right border of T-DNA. (**B**) First exon sequence containing target sites 1 and 2 for genome editing. R, W, and M are marked by IUPAC notation. Single nucleotide polymorphism (SNP) shows different bases between *EPH1La* and *EPH1Lb*. PAM, protospacer adjacent motif.

We attempted to produce *eph1l* mutants using the CRISPR/Cas9 system to investigate whether *EPH1L* was actually involved in flower senescence. Accordingly, we constructed a binary CRISPR/Cas9 vector, pSALS-35SpCas9-CmYLCVpEPH1Lt1t2, containing tandem single-guide RNAs (sgRNAs) that targeted the first exon of *EPH1L* (Figure 4). This binary vector was introduced to “Albireo” via *Agrobacterium*-mediated transformation, and the bispyribac-sodium-resistant lines were selected. After two rounds of infection, 28 lines of bispyribac-sodium-resistant lines were obtained. Sanger sequencing analysis revealed that 6 of the 28 lines obtained contained biallelic genome-edited sequences. Finally, three genome-edited lines (#6-6, #8-2, #8-5) were applied for the evaluation of flower longevity. Table 1 depicts the detailed sequence of each allele of *EPH1L* of #6-6, #8-2, and #8-5 lines. Insertions or deletions were noted in the target sequences of *EPH1L* in all three lines. These mutations generated premature stop codons that were predicted to produce non-functional partial EPH1L (Table 2), indicating that these were *eph1l*-knockout mutant lines. Furthermore, we performed RT-PCR analysis and detected the same sequences corresponding to the edited genomic sequences in each of the three lines. These results clearly showed that the functional knockout of *EPH1L* was achieved as in the case of the genes involved in flower color [21,22] and overwintering [35] in gentians.

### 2.5. Evaluation of Flower Longevity of eph1l-Genome-Edited Gentian Plants and the Involvement of EPH1L in Flower Senescence

**Figure 5:**
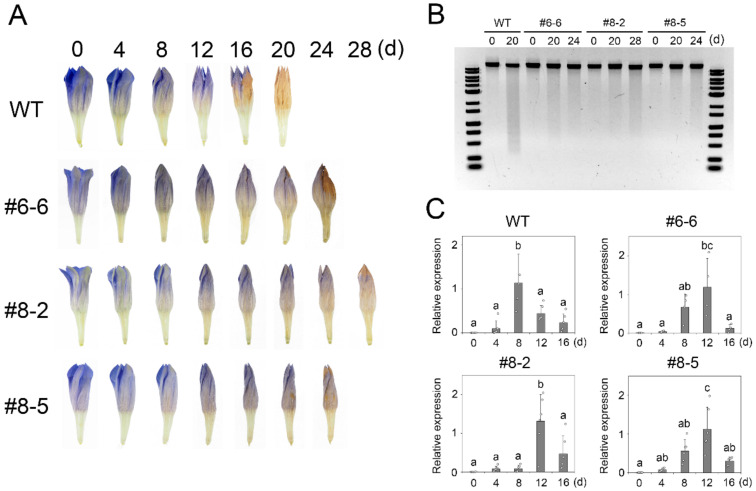
Evaluation of the flower longevity of *eph1l* genome-edited knockout mutant lines (#6-6, #8-2, and #8-5) lines. (**A**) Changes in corolla appearance every 4 days. (**B**) Image of the agarose gel electrophoresis of the genomic DNA from wild type (WT) and three *eph1l* lines before and after dark-induced senescence treatments. (**C**) The relative expression level of *SAG12* from WT and three *eph1l* lines determined by quantitative reverse transcription polymerase chain reaction (*n* = 4–6). Different letters indicate significant differences during the 16 d treatment period in each line (Tukey–Kramer test, *p* < 0.05).

To evaluate the flower longevity of genome-edited lines, #6-6, #8-2, and #8-5, we applied the dark-induced senescence system established here. Figure 5A depicts the change in the appearance of corollas of the *eph1l* mutants every 4 days. In the wild type (WT), the top of the corolla turned brown from the 16th day, and the whole corolla turned brown on the 20th day. On the other hand, no browning was noted on the 16th day in the corolla of all *eph1l* mutants. In addition, when the degradation levels of the genomic DNAs of the *eph1l* mutants were examined, the degradation levels at the 20th day after the start of the dark-induced senescence treatment were clearly suppressed relative to that of WT (Figure 5B). Comparing the 20th and 24th days of # 6-6, it looked almost unchanged, which was considered as due to the variability among flower samples, indicating that the level of senescence delay was variable. However, in both cases, the smear pattern was clearly thinner than that at the 20th day of WT. Furthermore, analysis of the expression levels of *SAG12* in the *eph1l*-knockout mutant lines by qRT-PCR clarified that the expression peak of *SAG12* was on the 12th day in all mutant lines, which was delayed when compared to those of WT (Figure 5C). All data obtained from the *eph1l*-knockout mutant lines demonstrated that these mutants had a prolonged flower longevity associated with delayed genomic DNA degradation and *SAG12* expression. Based on these results, *EPH1L* can be considered as one of the factors that accelerate the senescence of gentian flowers, and our results suggest that the *EPH1L* function is equivalent to that of the *EPH1* of Japanese morning glory.

Although numerous studies have been performed on ethylene-mediated flower senescence, there is little information available on ethylene-independent flower senescence. To date, an NAC transcription factor *EPH1* was reported to regulate the progression of PCD and be involved in petal senescence in Japanese morning glory, which demonstrated ethylene-independent petal senescence [8,36]. The NAC-type transcription factors are specific to plants and are involved in various processes, including plant development, cell division, wood formation, fruit maturation, and stress responses, as well as senescence [37,38,39,40,41,42].

The function of the NAC family in senescence has been extensively studied in *Arabidopsis thaliana*. Among the *NAC* genes belonging to the NAM subfamily, *ORE1* [43] and *ORS1* [44] have been reported to be involved in leaf senescence. However, *NAC* other than those belonging to the NAM subfamily, such as *NAP* [38] and *VNI2* [45], are involved in leaf senescence, which indicates that the NAM subfamily is not specialized for leaf senescence. Namely, it is believed that there are complicated pathways that promote senescence controlled by these transcription factors. *EPH1* and *EPH1L* belong to the NAM subfamily, and both have been shown to be involved in flower senescence. Further research is needed to clarify the mechanism of flower senescence regulation in gentians and other ornamental flowers.

Currently, the molecular mechanism of flower senescence mediated by the *EPH1* family is almost unknown. The relationship between ethylene signaling and EPH1L in gentian aging is also unclear. In the present study, “Albireo,” for which genome-editing technology has been established in gentians, was used as the material. “Albireo” is a cultivar derived from *G. scabra* and *G. triflora* with different ethylene sensitivities. Thus, it is not suitable as a material for analyzing the relationship between ethylene signaling and EPH1L in flower senescence. To clarify these issues, it is necessary to produce genome-edited lines for *G. scabra* and *G. triflora* and subject them to detailed analyses. In future research, we would like to address these issues and clarify the molecular mechanism of gentian flower senescence.

## 3. Materials and Methods

### 3.1. Plant Materials and Isolation of Genomic DNAs and RNAs

We used the Japanese cultivated gentian “Albireo” (*Gentiana scabra* × *Gentiana triflora*) as a WT in this study. The details of this cultivar are described in the manuscript by Tasaki et al. [22]. In this study, WT plants were grown in the fields of the Iwate Agricultural Research Center under natural conditions or in a greenhouse at the Iwate Biotechnology Research Center (IBRC). The genome-edited lines were grown in soil in pots under natural daylight in a closed greenhouse at the IBRC. We extracted the genomic DNAs and total RNAs from the plants by using the GenElute Plant Genomic DNA Miniprep Kit (Sigma-Aldrich, St Louis, MO, USA) and the RNeasy Mini kit (Qiagen, Valencia, CA, USA), respectively.

### 3.2. Cloning of the EPH1L cDNAs and Genomic Sequences from Gentians

A gene homologous to *EPH1* was searched using the published RNA-seq database of the Japanese gentian cultivar Hakuju [46], and a candidate sequence named *EPH1L* was obtained. Based on the Hakuju *EPH1L* sequence, we designed primers for *EPH1* in the regions corresponding to the 5’UTR and 3’UTR and attempted to clone *EPH1L* from “Albireo” by PCR (Figure S1). First-strand cDNA was synthesized with 500 ng of total RNA using the ReverTra Ace^®^ qPCR RT Master Mix with gDNA Remover (Toyobo, Osaka, Japan) as per the manufacturer’s protocol. The PCR conditions were 98 °C for 2 min, followed by 25 cycles of 98 °C for 30 s, 60 °C for 10 s, and 68 °C for 30 s. The PCR product was ligated into the pCR™Blunt II-TOPO^®^ vector (Thermo Fisher Scientific, Waltham, MA, USA). Plasmid DNA was extracted from a culture of transformed *E. coli* (DH5α) using the FastGene PlasmidMini Kit (NIPPON Genetics, Tokyo, Japan). The plasmid DNA was sequenced using the BigDye terminator ver. 1.1 Cycle Sequencing kit (Applied Biosystems, Foster City, CA, USA) and the ABI PRISM 3500 Genetic Analyzer (Applied Biosystems). Using the “Albireo” genomic DNA as a template, the *EPH1L* genomic sequences containing introns from the start codon to the stop codon were determined by the same procedure.

### 3.3. qRT-PCR Analysis

Total RNAs were extracted from the corolla lobes and first-strand cDNAs were synthesized as mentioned earlier. qRT-PCR was performed using the Luna Universal qPCR Master Mix (New England Biolabs, Ipswich, MA, USA) on the QuantStudio^TM^3 Real-Time PCR System (Thermo Fishier Scientific). The reaction mixtures comprised 10 µL of the master mix, 0.8 µM of each primer, 2 µL of cDNA, and DNase-free H_2_O (up to 20 µL). The PCR conditions were as follows: 95 °C for 10 min; 40 cycles of 95 °C for 15 s; and 60 °C for 1 min. A melting curve analysis was performed to verify the specificity and identity of the qRT-PCR products. The expression levels of the corresponding genes were calculated according to the methods described in Takahashi et al. [35]. The relative expression level of each gene was normalized to that of the gentian *Actin* gene, with the highest expression value as 1. Individual data points were then plotted. The primer sets against the target genes are listed in Supplementary Table S1.

### 3.4. Dark-Induced Senescence Treatment

Corollas at stage 4 of the greenhouse-grown “Albireo” were collected and immediately transferred to water containing 0.1% of the Plant Preservative Mixture™ (Plant Cell Technology, Northwest, DC, USA), and then incubated under continuous dark conditions at 25 °C until sampling. The sampling was performed every 4 days, and the samples were frozen in liquid nitrogen, lyophilized using a freeze dryer (FDU-2210, EYELA, TOKYO RIKAKIKAI CO, Tokyo, Japan), and then stored at −20 °C until further use.

### 3.5. Construction of a Binary Vector for Genome Editing and the Selection of Genome-Edited Gentian Plants

A binary CRISPR/Cas9 vector for targeting gentian *EPH1L* was constructed to harbor two single-guide RNA expression cassettes in our previous study. Two target sites in *EPH1L* exon 1 are shown in Figure 4. The resultant binary vector pSALS-35SpCas9-CmYLCVpEPH1Lt1t2 was transformed into the *A. tumefaciens* strain EHA101 by electroporation and used for gentian transformation. Plant transformation, transgenic plantlet selections, and mutation detection were performed with the same methods, as described by Takahashi et al. [35], except that the selection agent bispyribac-sodium was used. Briefly, after *Agrobacterium* infection, the leaf discs were placed on a half-strength solid MS medium containing 1.5% sucrose, 2.5 nM bispyribac-sodium, 200 mg/L cefotaxime, 10 mg/L meropenem, 5 mg/L thidiazuron, and 0.5 mg/L 1-naphthaleneacetic acid. The adventitious shoots regenerated from bispyribac-sodium-resistant calli were transferred to a half-strength solid MS medium containing 0.75 of 10 mg/L meropenem, and 3% sucrose for rooting. The validity of genome-edited plants was confirmed by Sanger sequencing analysis.

## 4. Conclusions

In this study, to clarify whether the gentian *EPH1L* is involved in flower senescence, we constructed *EPH1L* knockout mutant lines using the CRISPR/Cas9 system, and evaluated its flower longevity. As a result, similar to *EPH1*, gentian flower longevity is prolonged in *EPH1L* genome-edited knockout mutant lines, indicating that *EPH1L* accelerates flower senescence in gentians. Although functional analysis of the isolated gene is difficult, owing to the lack of mutants in most ornamental horticultural plants, this study clearly indicated the function of *EPH1L* in gentian flowers by using genome-editing technology. Further research is nonetheless warranted to elucidate how flower senescence is mediated by *EPH1L* in gentians. In addition, we also developed a new evaluation system for testing flower longevity using dark-induced senescence. We hope this system would be applied to various studies on flower longevity in the future.

## Figures and Tables

**Table 1:** Sequence analysis of *EPH1L* target sequences determined by Sanger sequencing.

Line	*EPH1L*	Target 1	In/del	Target 2	In/del
WT	*EPH1La*	ACAGAGCTGGAATTACCACCAGG	WT	CCTACTGATGAAGAACTAATTAC	WT
	*EPH1Lb*	ACAGAGCTGGAATTACCACCAGG	WT	CCTACTGATGAAGAACTAATTAC	WT
#6-6	*EPH1La*	ACAGAGCTGGAATTACC --------	−25 bp ^1^	------- GATGAAGAACTAATTAC	−25 bp ^1^
	*EPH1Lb*	ACAGAGCTGGAATTACCACCAGG	WT	CCTAC ---------- ATAATTAC	−10 bp
#8-2	*EPH1La*	ACAGAGCTGGAATTACC --------	−25 bp ^1^	------- GATGAAGAACTAATTAC	−25 bp ^1^
	*EPH1Lb*	ACAGAGCTGGAAT --- CACCAGG	−3 bp	CCTAC ----- AAGAACTAATTAC	−5 bp
#8-5	*EPH1La*	ACAGAGCTGGAATTA (256 bp insert) -- ACCAGG	+236 bp −2 bp	CCT ------- AAGAACTAATTAC	−7 bp
	*EPH1Lb*	ACAGAGCTGGAA ----- ACCAGG	−5 bp	CCT -------------- AATTAC	−14 bp

^1^ The sequence 3 bp upstream of the PAM sequences was excised. The red characters represent the PAM sequences.

**Table 2:** Amino acids sequences of WT and mutant forms of EPH1L.

Line	*EPH1L*	Deduced Amino Acids
WT	*EPH1La*	MEKNSEVSKPVESLETELELPPGFRFHPTDEELITHYLTPKVFDNSFSARAIGEVDL…
	*EPH1Lb*	MEKNSEVSKPVENLETELELPPGFRFHPTDEELITHYLTPKVFDYSFSARAIGEVDL…
#6-6	*EPH1La*	MEKNSEVSKPVESLETELELPMKN *
	*EPH1Lb*	MEKNSEVSKPVENLETELELPPGFRFHPT *
#8-2	*EPH1La*	MEKNSEVSKPVESLETELELPMKN *
	*EPH1Lb*	MEKNSEVSKPVENLETELESPGFRFHPTRTNYSLSHPKSFRLQLFCQSHWGGLEES *
#8-5	*EPH1La*	MEKNSEVSKPVESLETELELGI *
	*EPH1Lb*	MEKNSEVSKPVENLETELETRISFSSLLTISPQKFSTTAFLPEPLVRWT *

Asterisks indicate premature stop codons. The red characters represent the deduced mutated amino acid sequences.

## Data Availability

All data supporting the findings of this study are available within the paper and within its Supplementary Data published online.

## Supplementary Materials

The following supporting information can be downloaded at: https://www.mdpi.com/article/10.3390/ijms23105608/s1.

## Abbreviations

CRISPR/Cas9, clustered regularly interspaced short palindromic repeats/CRISPR-associated protein 9; *EPH1*, *EPHEMRAL1*; *EPH1L*, *EPEMERAL1-LIKE*; PCD, programmed cell death; qRT-PCR, quantitative reverse transcription polymerase chain reaction; *SAG12*, senescence-associated gene 12; sgRNAs, single-guide RNAs; STS, silver thiosulfate
