# Supplementary material for: Isolation and Functional Analysis of EPHEMERAL1-LIKE (EPH1L) Genes Involved in Flower Senescence in Cultivated Japanese Gentians
Source: Int J Mol Sci. 2022 May 17;23(10):5608. doi: 10.3390/ijms23105608 (PMC9147615; doi:10.3390/ijms23105608)
Supplement: Supplementary file 1 [file ijms-23-05608-s001.zip › ijms-1691055-supplementary.pdf]

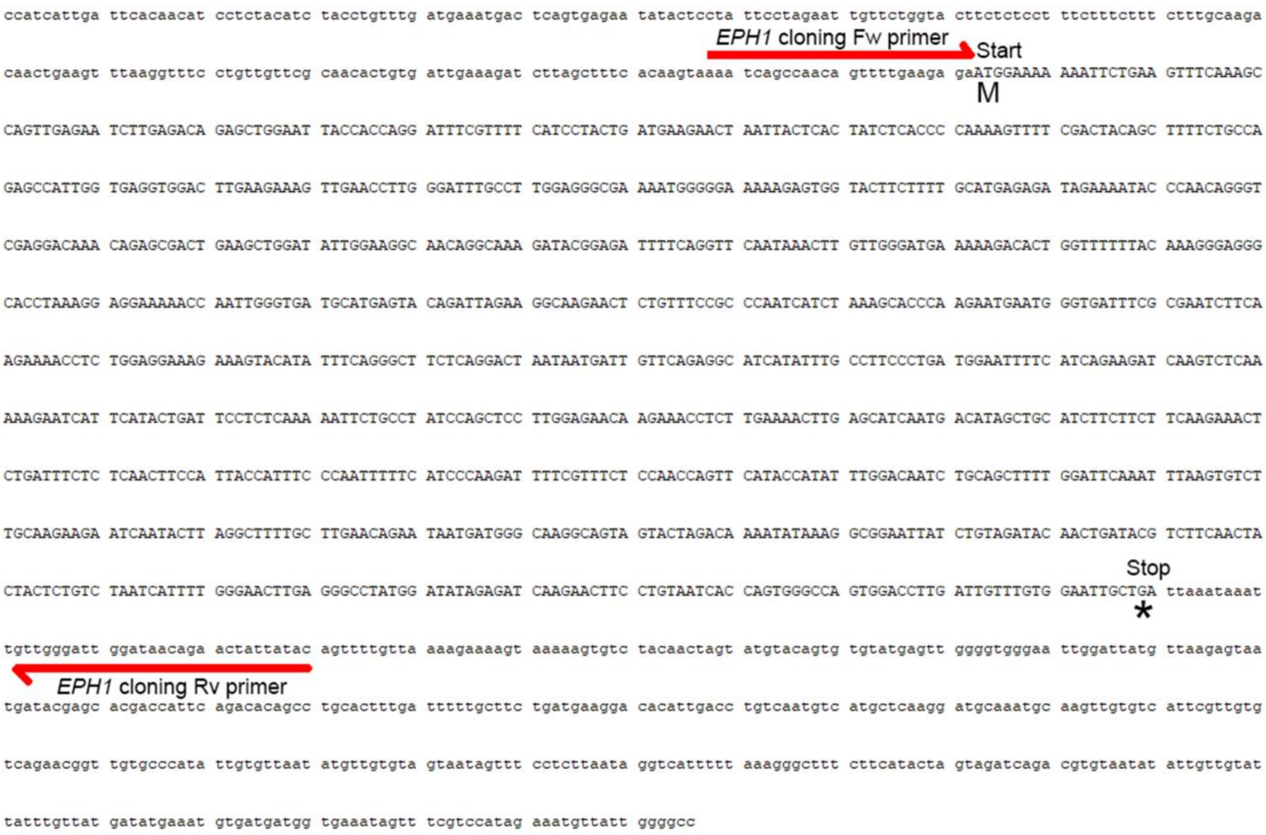

Figure S1. *EPH1* homologous sequence from RNA-seq database of the Japanese gentian “Hakuju (accession no. DRA012949).

EPH1La genomic sequence

ATGGAAAAAA ATTCTGAAGT TTCAAAGCCA GTTGAGAGTC TTGAGACAGA GCTGGAATTA CCACCAGGAT TTCGTTTTCA TCCTACTGAT GAAGAACTAA TTACTCACTA TCTCACCCCA

AAAGTTTTCG ACAACAGCTT TTCTGCCAGA GCCATTGGTG AGGTGGACTT GAAGAAAGTT GAACCTTGGG ATTTGCCTTg tgggtattct ttcattgcag caactatttt taatttctgg

gtttttttctt ttctgcagaa atttgattat tagatgttca aatatgctaa aaatctgaac tttttgttga tattttagGG AGGGCGAAAA TGGGGGAAAA AGAGTGGTAC TTCTTTTGCA

TGAGAGATAG AAAATACCCA ACAGGGTCGA GGACAAACAG AGCGACTGAA GCTGGATATT GGAAGGCAAC AGGGAAAGAT AAGGAGATTT TCAGGTTCAA TAAACTTGTT GGGATGAAAA

AGACACTGGT TTTTTACAAA GGGAGGGCAC CTAAAGGAGG AAAAACCAAT TGGGTGATGC ATGAGTACAG ATTAGAAGGC AAGAACTCTG TTTCCGCCCA ATCATCTAAA GCACCCAAGg

tgaagattca actctatgtc tatacaatta catatttttc ctctctgctc ccctgtattt catttgagta caacaaataa atggccatag cttatagtta tattacaggt ggtgaatttt

atggtgttct tgccttcttt tattcttctt tttctgcagA ATGAATGGGT GATTTCGCGA ATCTTCAAGA AAACCTCTGG AGGAAAGAAA GTACATATTT CAGGGCTTCT CAGGACTAAT

AATGATTGTT CAGAGGCATC ATATTTGCCT TCCCTGATGG AATTTTCATC AGAAGATCAA GTCTCAAAAA GAATCATTCA TACTGATTCC TCCTCCTCTA AAAAATTCTG CCTATCCAGC

TCCTTGGAGA ACAAGAAACC TCTTGAAAAC TTGAGCATCA ATGACATAGC TGCATCTTCT TCTTCAAGAA ACTCTGATTT CTCTCCACTT CCATTACCAT TTCCCAATTT TTCATCCCAA

GATTTTCGTT TCTCCAACCA GTTCATACCA TATTTGGACA ATCTGCAGCT TTTGGATTCA AATTCAAGTG TCTTGCAAGA AGAATCAATA CTTAGGCTTT TGCTTGAACA GAATAATGAT

GGGCAAGGCA GTAGTACTAG ACAAAATATA AAGGTGGAAT TATCTGTAGA TACAACTGAT ATGTCTTCAA CTACTACTCT GTCTAATCAT TTTGGGAACT TGAGGGCCTA TGGATATAGA

GATCAAGAAC TTCCTGTAAT CACCAGTGGG CCAGTAGACC TTGATTGTTT GTGGAATTGC TGA

EPH1Lb genomic sequence

ATGGAAAAAA ATTCTGAAGT TTCAAAGCCA GTTGAGAATC TTGAGACAGA GCTGGAATTA CCACCAGGAT TTCGTTTTCA TCCTACTGAT GAAGAACTAA TTACTCACTA TCTCACCCCA

AAAGTTTTCG ACTACAGCTT TTCTGCCAGA GCCATTGGTG AGGTGGACTT GAAGAAAGTT GAACCTTGGG ATTTGCCTTg tgggtattct ttcattgcag caactatttt taatttctgg

gtttttttatt ttctgcagaa atttgattat tagatgttca aatatgctaa aaatctgaac tttatgttga tattttagGG AGGGCGAAAA TGGGGGAAAA AGAGTGGTAC TTCTTTTGCA

TGAGAGATAG AAAATACCCA ACAGGGTCGA GGACAAACAG AGCGACTGAA GCTGGATATT GGAAGGCAAC AGGCAAAGAT ACGGAGATTT TCAGGTTCAA TAAACTTGTT GGGATGAAAA

AGACACTGGT TTTTTACAAA GGGAGGGCAC CTAAAGGAGG AAAAACCAAT TGGGTGATGC ATGAGTACAG ATTAGAAGGC AAGAACTCTG TTTCCGCCCA ATCATCTAAA GCACCCAAGg

tgaagattca actctatgtc tttataatta catatttttc ctctccgctc ccctgtattt catttgagta caacaaataa atggccatca ggccatagct tatagttata ttacaggtgg

tgaattttat ggtgttcttg ccttctttta ttcttctttt tctgcagAAT GAATGGGTGA TTTCGCGAAT CTTCAAGAAA ACCTCTGGAG GAAAGAAAGT ACATATTTCA GGGCTTCTCA

GGACTAATAA TGATTGTTCA GAGGCATCAT ATTTGCCTTC CCTGATGGAA TTTTCATCAG AAGATCAAGT CTCAAAAAGA ATCATTCATA CTGATTCCTC TCAAAAATTC TGCCTATCCA

GCTCCTTGGA GAACAAGAAA CCTCTTGAAA ACTTGAGCAT CAATGACATA GCTGCATCTT CTTCTTCAAG AAACTCTGAT TTCTCTCAAC TTCCATTACC ATTTCCCAAT TTTTCATCCC

AAGATTTTCG TTTCTCCAAC CAGTTCATAC CATATTTGGA CAATCTGCAG CTTTTGGATT CAAATTTAAG TGTCTTGCAA GAAGAATCAA TACTTAGGCT TTTGCTTGAA CAGAATAATG

ATGGGCAAGG CAGTAGTACT AGACAAAATA TAAAGGCGGA ATTATCTGTA GATACAACTG ATACGTCTTC AACTACTACT CTGTCTAATC ATTTTGGGAA CTTGAGGGCC TATGGATATA

GAGATCAAGA ACTTCCTGTA ATCACCAGTG GGCCAGTGGA CCTTGATTGT TTGTGGAATT GCTGA

Figure S2. Genomic sequences of *EPH1La* and *EPH1Lb* from the start codon to stop codon.

The green overlines represent the first and second introns of *EPH1Ls*.

Table S1 List of primers used in this study.

Primer name	Sequence (5' to 3')	Purpose
EPH1L cloning Fw	AAATCAGCCAACAGATTTTGAAGAG	Cloning
EPH1L cloning Rv	ATAGTTCTGTTATCCAATCCCAAC	Cloning
EPH1L Fw	ACCTTGGGATTTGCCTTGGA	qRT-PCR
EPH1L Rv	AGTACCACTCTTTTTCCCCCA	qRT-PCR
SAG12 Fw	CGTCGTTTTCTCTATCAAGTTTCAGAT	qRT-PCR
SAG12 Rv	TTCTTCTCTCTCCAATCCACACTTAG	qRT-PCR
Actin Fw	ACATTGTTCTCAGTGGTGGTTCA	qRT-PCR
Actin Rv	GGAGCCAAAGCAGTGATCTCTT	qRT-PCR
